# Learning policy lessons from the pandemic: the UK’s COVID Inquiry

**DOI:** 10.1093/eurpub/ckad222

**Published:** 2024-02-05

**Authors:** Martin McKee

**Affiliations:** Department of Health Services Research and Policy, London School of Hygiene & Tropical Medicine, London, UK

Faced with a novel virus, SARS-CoV-2, whose virulence and transmissibility was unknown, policymakers everywhere had to decide when and how to respond. It is unsurprising that many mistakes were made.

Next time, hopefully, it will be different. Scientific understanding of pandemic threats has increased enormously. Yet, as the failure of the Global Health Security Index to predict outcomes showed, technical preparedness was not enough.^1^ Functioning political structures and processes were crucial. Much has been written about the former but there has been much less on the latter.

The challenges of assessing the political aspects are formidable. Decision-making within governments takes place out of sight. It is often only when politicians write their biographies that we are able to see into the corridors of power, and then often from the perspective of the author, who may prioritize enhancing their reputation over reporting the truth.

Some outcomes may simply reflect who was in charge at the time. The USA response could have been even worse had Anthony Fauci not been there to limit the damage that an unconstrained Donald Trump could have done. There is some research on the association between pandemic outcomes and leaders with certain characteristics, such as women and populist leaders but there is less on how the structures and processes within governments worked or did not work.

Instead, we can look to the various inquiries being held. However, most, such as the Swedish COVID Commission, focused on the decisions that were made rather than how they were made. An exception was the inquiry by the French Senate that led, briefly, to the indictment of the health minister during the pandemic, although the lessons to be drawn were unclear. The same may be true of the recently announced inquiry by the Italian Parliament, given concerns that it will be used to settle political scores. The European Parliament has conducted an inquiry into the response of the European Commission but while the report expresses numerous concerns and makes many recommendations, it says little about what went wrong or how decisions were made. It has also been criticized for the secrecy of its proceedings.

In these circumstances, much attention has focused on the UK’s COVID Inquiry,^2^ by far the most extensive anywhere (there is another similar one in Scotland but it began somewhat later). Led by an experienced judge, its proceedings are in public. Witnesses, who can be compelled to attend, have provided detailed statements and a wealth of other material, including personal diaries, emails and WhatsApp messages. Many individuals, including scientists, civil servants and politicians, are being examined in sessions broadcast live, by lawyers acting on behalf of the Inquiry and other interested parties (core participants), such as organizations representing bereaved families.

So far, the Inquiry has not disappointed. Almost all those giving evidence concede that, had the initial lockdown taken place earlier, when there was already sufficient evidence to do so, many lives would have been saved. Equally, most accept that a summer 2020 decision to provide subsidies to those eating in restaurants caused the deaths of many more. Neither of these are surprises as the research was undertaken some time ago.^3^ The real contribution is revealing how and why key decisions were, or were not, made.

We now know that, at the onset of the pandemic, the machinery of government was in a state of utter chaos. Prime Minister Boris Johnson was struggling to reconcile his natural reluctance to impose rules that might restrict freedom with mounting evidence of the scale of the crisis. One of his senior advisers referred to him as the trolley, a reference to a supermarket trolley with a defective wheel that veered from side to side. His Director of Communications, argued that COVID-19 was ‘the wrong crisis for this Prime Minister’s skillset’. The government’s Chief Scientific Officer noted in his diary how the ‘PM is bamboozled’ and complained that ‘Watching PM get his head round stats is awful’.^4^

Key relationships were toxic. Johnson’s Permanent Secretary said, of those he was working with, that ‘It is like taming wild animals. Nothing in my past experience has prepared me for this madness’. The Cabinet Secretary said of the Health Minister ‘I'm fast losing confidence in Hancock’s candour as well as grip’ while one of the Prime Minister’s advisers said ‘He's lied his way through this and killed people…’. In his evidence, the Minister challenged how he was portrayed and, in return, provided evidence that his calls for early action had been ignored.

The situation was as bad in summer 2020, when the nature of the virus was much better understood and the then Finance Minister, now Prime Minister Rishi Sunak, launched his ill-fated restaurant subsidy. His statement to the Inquiry said that he heard no concerns from scientific advisers, yet those same advisers pointed out that the risks were obvious and they knew nothing of the scheme until it was announced. Sunak was overheard saying that ‘It is all about handling the scientists, not handling the virus’. Perhaps inevitably, the Inquiry is also highlighting the challenges of communicating scientific evidence to policymakers.

Inevitably, media attention has focused on politicians’ lack of understanding of basic science and questions about their honesty and humanity, as when Johnson said ‘let the bodies pile high’. But that misses the point. The Inquiry is revealing how the system of government in the UK is dysfunctional. This is hardly a revelation to many observers^5^ but now incontestable.

The UK, with its very strong scientific base and public health expertise should have done well in the pandemic. It did not. So were its political structures uniquely dysfunctional? Maybe, but it is at least plausible that similar problems can the found in some other countries. Unfortunately, until they conduct similarly probing inquiries, we may never know.

*Conflicts of interest*: M.M. has provided two witness statements to the Inquiry and is a member of Independent SAGE, a group of scientists that communicated science to the UK public throughout the pandemic. He is also Past President of the British Medical Association, which is a core participant in the Inquiry.

## Funding

No specific funding was involved.
